# Serum YKL-40 Levels Are Associated with the Atherogenic Index of Plasma in Children

**DOI:** 10.1155/2020/8713908

**Published:** 2020-09-26

**Authors:** Yoowon Kwon, Ju Hee Kim, Eun Kyo Ha, Hye Mi Jee, Hey Sung Baek, Man Yong Han, Su Jin Jeong

**Affiliations:** ^1^Departments of Pediatrics, CHA Bundang Medical Center, CHA University School of Medicine, Seongnam 13496, Republic of Korea; ^2^Department of Pediatrics, Kangnam Sacred Heart Hospital, Hallym University College of Medicine, Seoul 07441, Republic of Korea; ^3^Department of Pediatrics, Kandong Sacred Heart Hospital, Hallym University College of Medicine, Seoul 05355, Republic of Korea

## Abstract

YKL-40, also known as chitinase-3-like protein 1, is an inflammatory glycoprotein that is secreted by various cell types under acute, chronic, and subclinical inflammation conditions. Elevated serum YKL-40 levels are reportedly independently related to diabetes mellitus, coronary artery disease, acute myocardial infarction, and cardiovascular mortality in adults. Therefore, we aimed to investigate the relationship between serum YKL-40 levels, lipid abnormalities, and the atherogenic index of plasma (AIP) in children. We enrolled 479 children aged 10–12 years (mean age: 11.52) in this general population-based, cross-sectional study. All subjects completed questionnaires and were subjected to multifrequency bioelectrical impedance analysis (BIA) to measure their height, weight, and body mass index (BMI). We collected serum samples from all participants to measure YKL-40, total cholesterol (TC), low-density lipoprotein cholesterol (LDL-C), high-density lipoprotein cholesterol (HDL-C), and triglyceride (TG) levels. Mean serum YKL-40 levels were significantly higher in the low-HDL-C (*p* = 0.017) and high-TG (*p* = 0.010) groups but were not related to TC and LDL-C levels. YKL-40 levels were also higher in the high AIP group (*p* = 0.007). After adjusting for age, gender, and BMI *z*-score, the associations between serum YKL-40 levels and TG levels (*p* = 0.003), the TG-to-HDL-C ratio (*p* = 0.019), and the AIP value (*p* = 0.012) remained significant. Based on these findings, we suggest that serum YKL-40 may be a useful initial screening tool or follow-up risk indicator for lipid abnormalities, atherosclerosis, and cardiovascular disease in children and adolescents with risk factors, regardless of obesity.

## 1. Introduction

YKL-40 is a 40 kDa heparin- and chitin-binding glycoprotein, also known as chitinase-3-like protein 1 or human cartilage glycoprotein 39, which is secreted by various cells and holds proinflammatory activity [1–3]. Elevated circulating serum YKL-40 levels have been noted in many pathological conditions, such as acute and chronic inflammation, cancer, liver fibrosis in nonalcoholic fatty liver disease, insulin resistance, obesity, endothelial dysfunction, atherosclerosis, and even cardiovascular disease (CVD) [1–9].

Dyslipidemia is an abnormal amount of lipids—including total cholesterol (TC) and triglycerides (TG)—and their transporting lipoproteins, such as low-density lipoprotein cholesterol (LDL-C), high-density lipoprotein cholesterol (HDL-C), and very low-density lipoprotein cholesterol (VLDL-C), in the blood [10]. Accumulated serum lipids migrate to the subintimal layer where they constitute an important risk factor for the formation of atherosclerosis [11], and several studies have revealed that lipid metabolism abnormalities at a young age increase the long-term prevalence of CVD [12–14]. Thus, screening for dyslipidemia in children is essential to preventing future CVD risk.

Recently, the atherogenic index of plasma (AIP) value, which is calculated as the logarithmic transformation of the TG-to-HDL-C ratio, has been used not only as an optimal indicator of dyslipidemia but also as a strong novel index for the risk of atherosclerosis and CVD [11, 15]. However, fasting blood sampling is not always easy with children, which limits the assessment of the lipid profile and AIP values.

A previous study examined the relationship between serum YKL-40 levels, obesity, and insulin resistance in prepubertal children [5], but most other studies on the use of YKL-40 as a potential disease marker have been limited to adults. Therefore, we aimed to investigate the link between serum YKL-40 levels, lipid abnormalities, and AIP in children.

## 2. Materials and Methods

### 2.1. Subjects

This general population-based, cross-sectional study included children from 22 randomly selected classrooms from 11 elementary schools in Seongnam City (Korea) between March and August 2017 and was sponsored by the Seongnam City government [16]. The study was approved by the CHA University Institutional Review Board (2017-04-049). Written informed consent documents were obtained from all parents or guardians of the participating children. We enrolled 620 elementary school students aged 10–12 years whose parents submitted completed questionnaires. We reviewed the questionnaires to determine the population demographics. Serum samples to analyze YKL-40 levels were available from 479 of the 620 children, who were included in the final study sample. Participants who had been diagnosed with acute, chronic, or autoimmune diseases were excluded.

### 2.2. Anthropometric Measurements

The study participants were tested by multifrequency bioelectrical impedance analysis (BIA; InBody720, Biospace, Seoul, Korea) while wearing light indoor clothes and no shoes. Various parameters were automatically measured within 2 minutes, including body mass index (BMI), lean body mass, muscle fat ratio, body fat ratio, and abdominal fat ratio. We calculated the BMI of each subject based on their height and weight obtained by BIA. Standardized *z*-scores for sex and age were obtained using the LMS method described in the 2017 Korean National Growth Charts for children and adolescents [17, 18]. Overweight was defined as a BMI *z*-score of 1.04 (85^th^ percentile) or higher, and obesity was defined as a BMI *z*-score of 1.64 (95^th^ percentile) or higher.

### 2.3. Measurement of YKL-40

Venous serum samples were obtained and serum YKL-40 levels were measured in duplicate using a commercial Human Chitinase 3-like 1 Quantikine Immunoassay according to the manufacturer's instructions (R&D Systems, Minneapolis, MN, USA). Plasma samples were diluted 50-fold, and the results were multiplied by the dilution factor (50). The standard curve range for the assay was 62.5 to 4,000 pg/mL, and the mean minimum detectable dose was 3.55 pg/mL. The intra-assay and interassay precision coefficients of variation were 4.7% and 6.9%, respectively.

### 2.4. Measurement of Lipid Profile

To detect the TC, LDL-C, HDL-C, and TG levels, we obtained venous blood samples after 12 hours of overnight fasting. Blood samples were collected in separator tubes containing silica and a gel clot (Becton, Dickinson and Company, Franklin Lakes, NJ, USA) and centrifuged and analyzed within 2 hours. We defined acceptable, borderline, and high (or low for HDL-C) levels according to the National Heart, Lung, and Blood Institute (NHLBI) definitions. Acceptable levels were TC < 170 mg/dL, LDL − C < 110 mg/dL, HDL − C > 45 mg/dL, and TG < 90 mg/dL; borderline levels were 170 ≤ TC < 199 mg/dL, 110 ≤ LDL − C < 129 mg/dL, 40 ≤ HDL − C ≤ 45 mg/dL, and 90 ≤ TG < 129 mg/dL; and high (or low for HDL-C) levels were TC ≥ 200 mg/dL, LDL − C ≥ 130 mg/dL, HDL − C < 40 mg/dL, and TG ≥ 130 mg/dL [19]. Dyslipidemia was defined as elevated levels of TC (≥200 mg/dL), LDL-C (≥130 mg/dL), or TG (≥130 mg/dL), or some combination thereof, as well as low levels of HDL-C (<40 mg/dL).

The AIP was calculated according to the formula log(TG/HDL − C), where each concentration was expressed in mg/dL. Based on the results of previous studies on the association of AIP with CVD risks [11, 20, 21], we defined groups based on AIP value: the low AIP group had an AIP value < 0.1 and the high AIP group had an AIP value > 0.24.

### 2.5. Statistical Analysis

Descriptive statistics of the data were summarized as mean and 95% confidence interval for the variables with normal distribution or as median and interquartile range (IQR) for the values with non-normal distribution. As the logarithmic transformed YKL-40 levels had a normal distribution, we used analysis of covariance (ANCOVA) with a post hoc test (LSD method) to assess the relationships between the serum lipid panel (covariance) and YKL-40 levels, adjusting for age and gender. For continuous variables, we used generalized linear models with the gamma function to determine the beta value between YKL-40 levels and the lipid profile. Multivariable model was defined after adjusting for age, gender, BMI *z*-score, lean body mass, muscle fat ratio, and body fat ratio. The level of statistical significance was set at a p value ≤ 0.05. All statistical analyses were performed using IBM SPSS Statistics 25.0 (IBM, Armonk, NY, USA).

## 3. Results

### 3.1. Subject Characteristics

**Figure 1:**
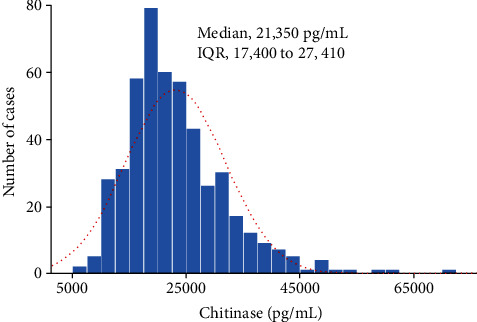
Serum concentrations of YKL-40 in the study population (*n* = 479).

A total of 479 children from the general population were enrolled in the study. The baseline demographic and clinical characteristics of the study group are shown in Table 1. The mean age of the group was 11.52 years and 49.9% (*n* = 239), of the subjects were male. Of the study group, 83.7% (*n* = 401) of the participants were of normal weight, 10.2% (*n* = 49) were overweight, and 6.1% (*n* = 29) were obese, based on their BMI *z*-scores. We calculated the mean and median values of the lipid profiles of the groups, including their TC, LDL-C, HDL-C, and TG levels.  Figure 1 shows the serum YKL-40 levels in the study population; the median serum YKL-40 level was 21,350 pg/mL.

### 3.2. Correlations between Serum YKL-40 Levels and Dyslipidemia and AIP

As summarized in Table 2, when the values were adjusted for age and gender, the levels of YKL-40 were not statistically related to TC and LDL-C levels. There was a significant negative correlation between mean serum YKL-40 and HDL-C levels (*p* = 0.017). We also observed a meaningful positive correlation between serum YKL-40 and TG levels (*p* = 0.010). The adjusted geometric mean (GM) of YKL-40 was higher in the group with the high TG-to-HDL-C ratio (tertile 3) than in the acceptable group (23,496 pg/mL vs 20,090 pg/mL; *p* < 0.001). In addition, YKL-40 levels were significantly higher in the high AIP group than in the acceptable group (22,542 pg/mL vs 20,183 pg/mL; *p* = 0.007) (Table 3).

### 3.3. Multivariate Linear Regression Analysis for Lipid Parameters and Serum YKL-40

We performed multivariate linear regression analyses to exclude confounding factors. As shown in Table 4, after adjusting for age, gender, and BMI *z*-score, the associations between serum YKL-40 and TG (*p* = 0.003), the TG-to-HDL-C ratio (*p* = 0.019), and AIP value (*p* = 0.012) remained significant.

## 4. Discussion

YKL-40 is a new inflammatory marker related not only to cancer and acute and chronic inflammatory conditions but also to subclinical inflammatory conditions such as atherosclerosis and insulin resistance. Moreover, it also promotes cell proliferation and differentiation and extracellular tissue remodeling in response to endothelial damage. YKL-40 is secreted by various cells, including neutrophils, activated macrophages, fibroblast-like synovial cells, arthritic chondrocytes, cancer cells, and vascular smooth muscle cells in atherosclerotic plaques [1–3]. Serum YKL-40 concentrations are not affected by gender or physical exercise and show no diurnal or long-term variation [3, 22]. Moreover, recent studies have reported that elevated serum YKL-40 levels are independently related to type 1 and type 2 diabetes mellitus (DM) [23] and the incidence and extent of coronary artery disease, acute myocardial infarction, and cardiovascular mortality [24–26].

Previous studies in adults have shown an association between circulating YKL-40 and TG levels in patients with type 2 DM [27] and stable coronary artery disease [28]. Most YKL-40 studies have been conducted in patients with existing disease. However, in this study, we measured serum YKL-40 levels and lipid profiles in children from the general population who are not in a known disease state. We demonstrated correlations between serum YKL-40 levels and low HDL-C levels, high TG levels, a high TG-to-HDL-C ratio, and a high AIP value. After adjusting for age, gender, and BMI *z*-score, the YKL-40 levels were significantly higher in groups with high TG levels, a high TG-to-HDL-C ratio, and a high AIP value. In addition, similar tendency was observed when we regarding the association between the lipid profile and serum YKL-40 after adjusting for lean body mass, muscle fat ratio, or body fat ratio instead of BMI *z*-score (Supplementary Table 1, 2, and 3). Our study demonstrated that YKL-40 may be useful as an indicator of lipid abnormalities and AIP in children.

Knowledge of the physiological functions of YKL-40 is still scarce, and the mechanism through which YKL-40 is elevated in the setting of low HDL-C or high TG levels is not clear. Previous studies have suggested that visceral adipose tissue could be the main source of YKL-40 in obesity [29], and increased macrophage infiltration from the visceral adipose tissue may be an important component of the chronic inflammatory response that drives the release of serum YKL-40 [2]. This may also explain the positive correlation between YKL-40 and the abdominal fat ratio after BMI correction in this study (*p* = 0.012, data not shown), which was consistent with other studies in which serum YKL-40 was closely related to the waist-to-hip ratio, but not the BMI [29, 30].

Dyslipidemia in children and adolescents predicts dyslipidemia in adulthood [31], and lipid abnormalities in the first few years of life are associated with the progression of atherosclerosis caused by endothelial damage [14, 32]. Moreover, dyslipidemia is an important modifiable risk factor for CVD. Therefore, it is essential to diagnose and manage dyslipidemia early because it begins in and persists from childhood and adolescence and acts as a major risk factor for CVD in adulthood. The new lipoprotein subfraction-based concept AIP was recently introduced to improve on the conventional measurement of lipid components. This index consists of atherogenic and protective lipoproteins that predict atherosclerosis or CVD risks better than conventional lipid components alone [11, 15]. Several studies confirmed that AIP values < 0.1 may be associated with a low risk of CVD, whereas values between 0.1 to 0.24 and >0.24 are associated with medium and high risks, respectively [11, 20, 21]; we used these criteria in our study. We found that serum YKL-40 levels in children have a statistically significant relationship with dyslipidemia and AIP, which is a novel marker for CVD or coronary atherosclerosis.

Interestingly, our study revealed relationships between serum YKL-40 levels and lipid abnormalities and AIP in children regardless of BMI. According to a study using the NHLBI criteria in 2012, 19.7% of Korean children and adolescents aged 10–18 years had at least 1 type of lipid abnormality. Hypercholesterolemia, and high LDL-C, low HDL-C, and high TG levels were observed 6.5%, 4.7%, 7.1%, and 10.1%, respectively, of children and adolescents [33]. The incidence of dyslipidemia is steadily increasing along with the incidence of obesity and metabolic syndrome. Changes in lifestyle and environment, weight gain, unhealthy diet, and lack of physical activity have caused higher rates of childhood dyslipidemia than in the past [14, 34]. According to current guidelines, children with a family history of CVD or with risk factors such as hypertension, smoking, DM, obesity, and chronic kidney disease are screened by measuring their lipid profile after fasting for at least 8–12 hours. However, in clinical cases, lipid abnormalities are often found by chance even in children who are not obese. These children may fail to receive a diagnosis of dyslipidemia if no other risk factors are present. Serum YKL-40 correlates well with high TG levels and AIP regardless of BMI, so it may be a useful initial screening tool for lipid abnormalities, atherosclerosis, and CVD, even in children who are not obese. In addition, measurement of YKL-40 has the advantage of no intraday variation, meaning that serum samples can be obtained from children at any time [3, 22].

This study is the first to demonstrate a positive correlation between YKL-40 levels and TG levels, TG-to-HDL ratio, and AIP value in a large cohort of Korean children. However, some limitations should be noted. First, this study was a cross-sectional study with no prospective follow-up study; therefore, we cannot definitively confirm a relationship between serum YKL-40 levels, lipid abnormalities, and AIP. Second, it is not possible to be certain that children with unrecognized subclinical infections were fully excluded. We tried our best to exclude participants who had been diagnosed with acute, chronic, or autoimmune diseases; however, we could not confirm all inflammation-related conditions, such as atypical infection or a subclinical state of a not diagnosed disease. Finally, data on other confounding factors, such as lifestyle, diet, and hormonal levels, were not included in this analysis. Since serum lipids and lipoproteins are affected by various factors [35], we cannot rule out the possibility that these factors could impact on the relationship between YKL-40, lipid profiles, and AIP. It will be necessary to address these factors through further research. In addition, longitudinal follow-up study on the actual disease manifestation according to serum YKL-40 levels is still needed in order to expand our findings.

## 5. Conclusions

Serum YKL-40 levels significantly positively correlated with TG levels (*p* = 0.003), the TG-to-HDL-C ratio (*p* = 0.019), and the AIP value (*p* = 0.012) after adjusting for age, gender, and BMI *z*-score, but not with TC and LDL-C levels. Therefore, serum YKL-40 levels can strongly indicate lipid abnormality status and AIP without the need for a fasting blood sample for lipid profiling. Based on these findings, we suggest that serum YKL-40 may be a useful initial screening tool or follow-up risk indicator for lipid abnormalities, atherosclerosis, and CVD in children and adolescents with risk factors, regardless of obesity.

## Figures and Tables

**Table 1:** Demographic and clinical characteristics of the participants (*N* = 479).

Parameters	Values
Demographics	
Age, years, mean (95% CI)	11.52 (11.45–11.60)
Sex, male, *n* (%)	239 (49.9)
Anthropometrics	
Height, cm, mean (95% CI)	149.46 (148.75–150.17)
BMI *z* score, mean (95% CI)	-0.033 (-0.0126–0.061)
Normal, *n* (%)	401 (83.7%)
Overweight, *n* (%)^∗^	49 (10.2%)
Obesity, *n* (%)^∗^	29 (6.1%)
Lean body mass, median (IQR)	32.05 (28.80–36.00)
Muscle fat ratio, median (IQR)	3.20 (2.29–4.59)
Body fat ratio, median (IQR)	22.70 (17.00–29.10)
Blood lipid measurements	
TC, mg/dL, mean (95% CI)	169.24 (166.89–171.59)
LDL-C, mg/dL, median (IQR)	97.00 (82.00–112.50)
HDL-C, mg/dL, median (IQR)	58.00 (50.00–65.00)
TG, mg/dL, median (IQR)	107.00 (78.00–142.50)

Abbreviation: BMI, body mass index; CI, confidence interval; HDL-C, high-density lipoprotein cholesterol; IQR, interquartile range; LDL-C, low-density lipoprotein cholesterol; TC, total cholesterol; TG, triglycerides. Missing data in prematurity, *n* = 8; blood lipid measurements, *n* = 2. ^∗^Overweight is defined as BMI *z*-score over 1.04 (85th percentile), and obesity is defined as BMI *z*-score over 1.64 (95th percentile).

**Table 2:** Correlation between serum YKL-40 levels and dyslipidemia.

		Crude and adjusted GM		
Blood lipid level	Number	Crude GM (95% CI)	Adjusted GM (95% CI)^∗∗∗^	p-value
TC				
Acceptable (<170)	248	21,350 (20,361–22,382)	21,330 (20,417–22,335)	0.404^∗^
Borderline (170-199)	165	22,233 (21,095–23,426)	22,233 (21,037–23,496)	0.267^∗∗^
High (≥200)	64	20,950 (19,146–22,929)	20,892 (19,142–22,855)	0.681^∗∗^
LDL-C				
Acceptable (<110)	329	21,423 (20,582–22,299)	21,428 (20,606–22,284)	0.692^∗^
Borderline (110-129)	103	22,233 (20,758–23,817)	22,181 (20,701–23,823)	0.401^∗∗^
High (≥130)	45	21,414 (18,539–23,724)	21,428 (19,230–23,823)	0.979^∗∗^
HDL-C				
Acceptable (>45)	415	21,296 (20,563–22,059)	**21,281 (20,558**–**22,029)**	**0.037** ^∗^
Borderline (40-45)	41	22,714 (20,663–24,963)	22,750 (20,370–25,409)	0.264^∗∗^
Low (<40)	21	25,745 (21,266–31,167)	**25,822 (22,130**–**30,199)**	**0.017** ^∗∗^
TG				
Acceptable (<90)	184	21,071 (19,971–22,233)	**21,086 (19,998**–**22,181)**	**0.011** ^∗^
Borderline (90-129)	147	20,615 (19,457–21,887)	20,606 (19,408–21,777)	0.568^∗∗^
High (≥ 130)	146	23,339 (22,069–24,683)	**23,388 (22,029**–**24,774)**	**0.010** ^∗∗^

Abbreviation: CI, confidence interval; GM, geometric mean; HDL-C, high-density lipoprotein cholesterol; LDL-C, low-density lipoprotein; TC, total cholesterol; TG, triglyceride. ^∗^*p* values designate differences of covariance (ANCOVA) between three groups. ^∗∗^*p* values of the comparison between borderline or high groups with the healthy group by ANCOVA with post hoc analysis (LSD method). ^∗∗∗^Geometric mean and 95% confidence intervals of YKL-40 were calculated by ANCOVA adjusting for gender (boy and girl) and age with LSD method. Numbers in bold indicate significant differences (*p* < 0.05).

**Table 3:** Correlation between serum YKL-40 levels and AIP.

		Crude and adjusted GM		
	Number	Crude GM (95% CI)	Adjusted GM (95% CI)^∗∗∗^	*p* value
Ratio with TG to HDL-C				
Low (Tertile 1, <1.4)	158	20,109 (19,028–21,247)	**20,090 (19,010**–**21,281)**	**0.001** ^∗^
Borderline (Tertile 2, 1.4–2.2)	157	21,310 (20,109–22,583)	21,291 (20,137–22,542)	0.159^∗∗^
High (Tertile 3, ≥2.2)	162	23,447 (22,192–24,779)	**23,496 (22,233**–**24,831)**	**<0.001** ^∗∗^
AIP				
Low (<0.1)	123	20,211 (18,971–21,532)	**20,183 (18,923**–**21,527)**	**0.018** ^∗^
Borderline (0.1–0.24)	101	21,091 (19,678–22,599)	21,086 (19,633–22,594)	0.113^∗∗^
High (>0.24)	253	22,516 (21,512–23,561)	**22,542 (21,527**–**23,550)**	**0.007** ^∗∗^

Abbreviation: CI: confidence interval; GM: geometric mean; HDL-C: high-density lipoprotein cholesterol; TG: triglycerides. ^∗^*p* values designate difference of covariance between three groups; ^∗∗^*p* values of the comparison between borderline and high groups with the healthy group by analysis of covariance (ANCOVA) with post hoc analysis (LSD method) ^∗∗∗^Geometric mean and 95% confidence intervals of YKL-40 calculated by ANCOVA and adjusting for gender (boy and girl) and age with LSD method. Numbers in bold indicate a significant differences (*p* < 0.05).

**Table 4:** Multivariate linear regression analysis for lipid parameters with serum YKL-40.

	Concentration of serum YKL-40			
Variables^‡^	Unadjusted		Multivariable adjusted^∗^	
	*β* (95% CI)	*p* value	a*β* (95% CI)	*p* value
Total cholesterol	0.015 (-0.451–0.482)	0.948	0.022 (-0.438–0.482)	0.925
LDL-C	0.057 (-0.236–0.351)	0.702	-0.004 (-0.298–0.288)	0.979
HDL-C	**-0.420 (-0.759**–**-0.082)**	**0.015**	-0.221 (-0.590–0.148)	0.240
Triglyceride	**0.246 (0.095**–**0.398)**	**0.001**	**0.204 (0.048**–**0.360)**	**0.003**
TG to HDL-C ratio	**0.030 (0.011**–**0.049)**	**0.002**	**0.023 (0.004**–**0.043)**	**0.019**
AIP (log TG to HDL-C ratio)	**0.209 (0.088**–**0.329)**	**0.001**	**0.164 (0.036**–**0.292)**	**0.012**

Abbreviation: AIP: atherogenic index of plasma; aOR: adjusted odds ratio; CI: confidence interval; HDL-C: high-density lipoprotein cholesterol; LDL-C: low-density lipoprotein cholesterol; TG: triglyceride. ^∗^Outcomes derived from generalized linear regression analysis with gamma function for the concentration of YKL-40 associated with individual lipid profile level as a continuous variable, adjusting with age, gender, and BMI *z*-score. ^‡^Log transformed for lipid profiles. Numbers in bold indicate a significant difference (*p* < 0.05).

## Data Availability

The data used to support the findings of this study are available from the corresponding author upon request.

## Supplementary Materials

Supplementary Materials Supplementary material includes three supplementary tables showing the association between the lipid profile and serum YKL-40 after adjusting for lean body mass, muscle fat ratio, or body fat ratio instead of BMI *z*-score.
