# Supplementary material for: Serum YKL-40 Levels Are Associated with the Atherogenic Index of Plasma in Children
Source: Mediators Inflamm. 2020 Sep 26;2020:8713908. doi: 10.1155/2020/8713908 (PMC7533750; doi:10.1155/2020/8713908)
Supplement: Supplementary Materials — Supplementary material includes three supplementary tables showing the association between the lipid profile and serum YKL-40 after adjusting for lean body mass, muscle fat ratio, or body fat ratio instead of BMI z-score. [file 8713908.f1.docx]

Supplementary Table 1: Multivariate linear regression analysis for lipid parameters with serum YKL-40

|  | Concentration of serum YKL-40 | | | | | |
| --- | --- | --- | --- | --- | --- | --- |
| Variables^‡^ | Unadjusted |  | | Multivariable adjusted^*^ | |  |
|  | β (95% CI) | *P* value |  | aβ (95% CI) | *P* value | |
| Total cholesterol | 0.015 (-0.451 to 0.482) | 0.948 |  | 0.090 (-0.382 to 0.562) | 0.709 | |
| LDL-C | 0.057 (-0.236 to 0.351) | 0.702 |  | 0.080 (-0.216 to 0.375) | 0.598 | |
| HDL-C | **-0.420 (-0.759 to -0.082)** | **0.015** |  | **-0.372 (-0.725 to -0.020)** | **0.038** | |
| Triglyceride | **0.246 (0.095 to 0.398)** | **0.001** |  | **0.245 (0.091 to 0.398)** | **0.002** | |
| TG to HDL-C ratio | **0.030 (0.011 to 0.049)** | **0.002** |  | **0.029 (0.010 to 0.048)** | **0.003** | |
| AIP [Log TG to HDL-C ratio] | **0.209 (0.088 to 0.329)** | **0.001** |  | **0.204 (0.080 to 0.327)** | **0.001** | |

Abbreviation: *aOR*, adjusted odds ratio; *CI*, confidence interval; LDL-C, low-density lipoprotein cholesterol; HDL-C, high-density lipoprotein cholesterol; TG, triglyceride; AIP, atherogenic index of plasma. ^*^Outcomes derived from generalized linear regression analysis with gamma function for the concentration of YKL-40 associated with individual lipid profile level as a continuous variable, adjusting with age, gender and Lean body mass. ^‡^Log transformed for lipid profiles. Numbers in bold indicate a significant difference (*P* < 0.05).

Supplementary Table 2: Multivariate linear regression analysis for lipid parameters with serum YKL-40

|  | Concentration of serum YKL-40 | | | | | | |
| --- | --- | --- | --- | --- | --- | --- | --- |
| Variables^‡^ | Unadjusted |  | | Multivariable adjusted^*^ | | |  |
|  | β (95% CI) | *P* value |  |  | aβ (95% CI) | *P* value | |
| Total cholesterol | 0.015 (-0.451 to 0.482) | 0.948 |  |  | -0.078 (-0.539 to 0.382) | 0.739 | |
| LDL-C | 0.057 (-0.236 to 0.351) | 0.702 |  |  | -0.047 (-0.341 to 0.247) | 0.754 | |
| HDL-C | **-0.420 (-0.759 to -0.082)** | **0.015** |  |  | -0.279 (-0.630 to 0.071) | 0.118 | |
| Triglyceride | **0.246 (0.095 to 0.398)** | **0.001** |  |  | **0.198 (0.042 to 0.355)** | **0.013** | |
| TG to HDL-C ratio | **0.030 (0.011 to 0.049)** | **0.002** |  |  | **0.024 (0.004 to 0.043)** | **0.016** | |
| AIP [Log TG to HDL-C ratio] | **0.209 (0.088 to 0.329)** | **0.001** |  |  | **0.166 (0.039 to 0.292)** | **0.010** | |

Abbreviation: *aOR*, adjusted odds ratio; *CI*, confidence interval; LDL-C, low-density lipoprotein cholesterol; HDL-C, high-density lipoprotein cholesterol; TG, triglyceride; AIP, atherogenic index of plasma. ^*^Outcomes derived from generalized linear regression analysis with gamma function for the concentration of YKL-40 associated with individual lipid profile level as a continuous variable, adjusting with age, gender and muscle fat ratio. ^‡^Log transformed for lipid profiles. Numbers in bold indicate a significant difference (*P* < 0.05).

Supplementary Table 3: Multivariate linear regression analysis for lipid parameters with serum YKL-40

|  | Concentration of serum YKL-40 | | | | | |
| --- | --- | --- | --- | --- | --- | --- |
| Variables^‡^ | Unadjusted |  | | Multivariable adjusted^*^ | |  |
|  | β (95% CI) | *P* value |  | aβ (95% CI) | *P* value | |
| Total cholesterol | 0.015 (-0.451 to 0.482) | 0.948 |  | -0.048 (-0.514 to 0.417) | 0.839 | |
| LDL-C | 0.057 (-0.236 to 0.351) | 0.702 |  | -0.005 (-0.302 to 0.291) | 0.973 | |
| HDL-C | **-0.420 (-0.759 to -0.082)** | **0.015** |  | **-0.379 (-0.727 to -0.031)** | **0.033** | |
| Triglyceride | **0.246 (0.095 to 0.398)** | **0.001** |  | **0.235 (0.079 to 0.390)** | **0.003** | |
| TG to HDL-C ratio | **0.030 (0.011 to 0.049)** | **0.002** |  | **0.028 (0.009 to 0.048)** | **0.004** | |
| AIP [Log TG to HDL-C ratio] | **0.209 (0.088 to 0.329)** | **0.001** |  | **0.200 (0.075 to 0.325)** | **0.002** | |

Abbreviation: *aOR*, adjusted odds ratio; *CI*, confidence interval; LDL-C, low-density lipoprotein cholesterol; HDL-C, high-density lipoprotein cholesterol; TG, triglyceride; AIP, atherogenic index of plasma. ^*^Outcomes derived from generalized linear regression analysis with gamma function for the concentration of YKL-40 associated with individual lipid profile level as a continuous variable, adjusting with age, gender and body fat ratio. ^‡^Log transformed for lipid profiles. Numbers in bold indicate a significant difference (*P* < 0.05).
